# Active Targeting to Osteosarcoma Cells and Apoptotic Cell Death Induction by the Novel Lectin *Eucheuma serra* Agglutinin Isolated from a Marine Red Alga

**DOI:** 10.1155/2012/842785

**Published:** 2012-12-27

**Authors:** Keita Hayashi, Peter Walde, Tatsuhiko Miyazaki, Kenshi Sakayama, Atsushi Nakamura, Kenji Kameda, Seizo Masuda, Hiroshi Umakoshi, Keiichi Kato

**Affiliations:** ^1^Division of Chemical Engineering, Graduate School of Engineering Science, Osaka University, 1-3 Machikaneyama-cho, Toyonaka 560-8531, Osaka, Japan; ^2^Department of Materials, ETH, Wolfgang-Pauli-Straße 10, 8093 Zürich, Switzerland; ^3^Department of Pathogenomics, Graduate School of Medicine, Ehime University, Shitsukawa, Toon 791-0295, Ehime, Japan; ^4^Department of Bone and Joint Surgery, Graduate School of Medicine, Ehime University, Shitsukawa, Toon 791-0295, Ehime, Japan; ^5^Integrated Center for Science Shigenobu Station, Ehime University, Shitsukawa, Toon 791-0295, Ehime, Japan; ^6^Department of Materials Science and Biotechnology, Graduate School of Science and Engineering, Ehime University, 3 Bunkyo-cho, Matsuyama 790-8577, Ehime, Japan

## Abstract

Previously, we demonstrated that the novel lectin *Eucheuma serra* agglutinin from a marine red alga (ESA) induces apoptotic cell death in carcinoma. We now find that ESA induces apoptosis also in the case of sarcoma cells. First, propidium iodide assays with OST cells and LM8 cells showed a decrease in cell viability after addition of ESA. With 50 *μ*g/ml ESA, the viabilities after 24 hours decreased to 54.7 ± 11.4% in the case of OST cells and to 41.7 ± 12.3% for LM8 cells. Second, using fluorescently labeled ESA and flow cytometric and fluorescence microscopic measurements, it could be shown that ESA does not bind to cells that were treated with glycosidases, indicating importance of the carbohydrate chains on the surface of the cells for efficient ESA-cell interactions. Third, Span 80 vesicles with surface-bound ESA as active targeting ligand were shown to display sarcoma cell binding activity, leading to apoptosis and complete OST cell death after 48 hours at 2 *μ*g/ml ESA. The findings indicate that Span 80 vesicles with surface-bound ESA are a potentially useful drug delivery system not only for the treatment of carcinoma but also for the treatment of osteosarcoma.

## 1. Introduction

Osteosarcoma has one of the worst prognosis among all malignant tumors. Before 1970, the 5-year survival rate of the treated patients was only about 20% [1, 2]. The treatment of osteosarcoma currently involves surgical resection in combination with neoadjuvant chemotherapy. Despite advances in the neoadjuvant chemotherapy and in limb-salvage surgery, the disease-free survival rate still remains poor for patients with metastatic, recurrent, or unresectable osteosarcoma. Thus, novel selective therapeutic approaches against osteosarcoma are highly required.

Previously, we found that the novel lectin *Eucheuma serra* agglutinin (ESA), which was successfully isolated by Kawakubo et al. [3] from the marine red alga *Eucheuma serra*, specifically binds to carcinoma cell lines of human adenocarcinoma, human cervical squamous cell carcinoma, and marine adenocarcinoma but not to normal human fibroblasts or lymphocytes [4]. We also revealed, that the specific binding of ESA to carcinoma cells is based on specific interactions between ESA and the unique sugar chains of high mannose type on the surface of the carcinoma cells [4]. In a more recent study, Hori et al. [5] investigated the specific interactions between ESA and various unique sugar chains of high mannose type in detail. Furthermore, we successfully elaborated the basis for a novel type of drug delivery system (DDS) for cancer therapy using ESA (i) as targeting ligand to carcinoma tumors and (ii) as inducer of apoptosis due to specific ESA binding to carcinoma cells [6]. Recently, the general potential of certain types of sugar binding proteins (lectins) as promising, alternative antitumor drugs has been emphasized [7]. The antitumor activity of these lectins might be related directly to specific intermolecular interactions between the lectins and the sugar chains on the tumor cell surface [8]. However, whether lectins also have antitumor activities against osteosarcomas has not been clarified yet.

Generally, carcinomas which originate in epithelial cells and sarcomas which originate in mesenchymal cells (e.g., osteosarcoma) are thought to be quite different in their tumorigenesis as well as in the phenotypes including cytoskeleton, binding molecules, proliferation procedure, and surface glycoproteins [9, 10]. Therefore, different therapeutic approaches have been employed for the treatment of sarcomas, if compared with the therapies applied for the treatment of carcinomas, except for the surgical treatment. On the other hand, the existence of cell surface-bound sugar chain structures, which are common among carcinomas and sarcomas, but not present in normal cells, has been suggested [11]. Moreover, the concept of epithelial-mesenchymal transition in tumors implies common structures and/or mechanism among carcinomas and sarcomas [12, 13]. Therefore, on the basis of our previous *in vitro* and *in vivo* studies with ESA bound to Span 80 vesicles for targeting carcinoma cells [6], we found it worthwhile to investigate whether the lectin ESA can also be applied in a therapeutic approach against osteosarcomas. Span 80 is generally known in the food and cosmetic industries as sorbitan monooleate, although commercial Span 80 is a heterogeneous mixture of sorbitan mono-, di-, tri-, and tetra-esters [14]. We have already demonstrated that nonionic vesicles prepared from Span 80 have promising physicochemical properties (high membrane fluidity with temperature dependent fusiogenicity) which make this type of vesicle an attractive possible alternative to the commonly used liposomes *in vitro* and *in vivo* [6, 14–22].

Aim of the work was to clarify the specificity of the binding of ESA to either OST cells or LM8 cells, both being osteosarcoma cell lines. Furthermore, the potential effectivity of ESA as ligand on the surface of Span 80 vesicles [6, 14, 18, 19, 21, 22] with targeting function and as possible apoptosis-inducer for the treatment of osteosarcoma was also examined. In the work presented, the interactions between ESA and OST cells and between ESA and LM8 cells were examined by means of fluorescence microscopy and flow cytometry. As a result of our study, the evidence is presented that ESA specifically binds to these two types of osteosarcoma cells, followed by induction of apoptosis due to this specific ESA binding to the cells. Furthermore, we could demonstrate that ESA has a considerable potential as novel type of ligand immobilized onto PEGylated Span 80 vesicles, an important step towards the potential development of a therapy for the treatment of refractory osteosarcoma, as novel lipidic microcapsule drug-delivery system (DDS) for transporting and delivering anticancer drugs for the treatment of cancer [6].

## 2. Material and Methods

### 2.1. Materials

*Eucheuma serra* agglutinin (ESA) was extracted from the red alga *Eucheuma serra*, by means of ethanol precipitation, followed by purification with fast protein liquid chromatography (FPLC), using a 10 mmol/L sodium phosphate buffer (pH = 7.4) [3]. Propidium iodide (PI), *α*-mannnosidase, *β*-mannnosidase, endoglycosidase H, and rhodamine 6G were obtained from Sigma-Aldrich (St. Louis, MO, USA). The “Annexin V-PE Apoptosis Detection Kit I” which contains Annexin V-PE and 7-amino-actinomycin D (7-ADD) was obtained from Becton Dickinson Biosciences (Franklin Lakes, NJ, USA). The caspase assay system was purchased from Promega (Madison, WI, USA). Fluorescein isothiocyanate, isomer I (FITC), Span 80, cholesterol, and lecithin from soybeans were obtained from Wako Pure Chemical Industries (Osaka, Japan). The lecithin was purified by acetone precipitation [23]. The phospholipid 1,2-dioleoyl-*sn*-glycero-3-phosphoethanolamine-N-(succinyl) (SuPE) was obtained from Avanti Polar Lipids (Alabaster, AL, USA). DSPE-PEG_2000_ was from NOF Corporation (Tokyo, Japan). PBS (phosphate buffered saline) was composed of 137 mM NaCl, 2.7 mM KCl, 10 mM Na_2_HPO_4_ and 2 mM KH_2_PO_4_, (pH = 7.4).

### 2.2. Cells and Cell Cultures

Human osteosarcoma Takase (OST) cells were offered by Dr. Katsuro Tomita (Department of Orthopaedic Surgery, Kanazawa University School of Medicine, Japan), cultured in either ERDF medium (Kyokuto Pharmaceutical Industrial, Tokyo, Japan) or Dulbecco's Modified Eagle Medium (D-MEM) (Wako Pure Chemical Industries, Osaka, Japan) supplemented with 10% of fetal bovine serum (FBS) at 37°C in a humidified atmosphere consisting of 5% CO_2_. Murine osteosarcoma cell line (LM8 cells) was obtained from RIKEN (RIKEN BRC Cell Bank). These LM8 cells were grown in D-MEM supplemented with 10% of FBS at 37°C in a humidified atmosphere consisting of 5% CO_2_.

### 2.3. Cell Viability Assay

OST cells and LM8 cells were inoculated in 6-well culture plates at a cell density of 2.0 × 10^5^ cells/mL suspended in D-MEM with 10% FBS. After 16 hours, the medium in each plate was exchanged with 10% FBS D-MEM containing various concentration of ESA. After incubation during one day, the cell number and the viabilities of both types of cells were evaluated by means of the “Propidium Iodide Nucleic Acid Stain” using flow cytometry [24]. The viability assay of OST cells for EPV was also performed by the same way as above. In a similar way, time-courses of the viability of both OST cells and LM8 cells were experimentally measured in medium with ESA at a concentration of 50 *μ*g/mL.

### 2.4. Apoptosis Assay

Apoptosis was analyzed by using the “Annexin V-PE Apoptosis Detection Kit I” according to a previously published protocol [25–27]. OST cells or LM8 cells, at a concentration of 2 × 10^5^ cells/mL, were suspended in D-MEM containing 10% FBS, and then inoculated in 6-well culture plates. After 16 hours inoculation, the medium in each plate was exchanged with 10% FBS, D-MEM containing 50 *μ*g/mL ESA. The cell lines in each plate were incubated for different time periods, followed by twice washing with cold PBS. Using a cell counter, the washed cells were diluted to a concentration of 1 × 10^6^ cells/mL by resuspending the cells in 0.1 M Hepes/NaOH (pH 7.4) containing 1.4 M NaCl and 25 mM CaCl_2_ (“binding buffer”). Volumes of 100 *μ*L of the cell suspensions were transferred to 1.5 mL Eppendorf tubes. Solutions of 5 *μ*L of AnnexinV-PE and 5 *μ*L of 7-ADD were added to the suspensions, followed by vortexing and incubation for 15 min at room temperature in the dark. Then, 400 *μ*L “binding buffer” were added to each tube containing the incubated suspension, followed by analysis with a flow cytometer.

### 2.5. Caspase-3 Assay

Caspase-3 activity was evaluated spectrophotometrically at *λ* = 405 nm with the caspase-3 substrate Ac-DEVD-pNA. OST cells were suspended at 2.0 × 10^5^ cells/mL in D-MEM with 10% FBS and then pipetted into 6-well culture plates. After 16 hours of incubation at 37°C and 5% CO_2_, the medium in each plate was exchanged by 10% FBS, D-MEM containing either 50 *μ*g/mL ESA, or 50 *μ*g/mL ESA + ZVAD-FMK (=N-Benzyloxycarbonyl-Val-Ala-Asp(O-Me) fluoromethyl ketone) which is a known caspase-3 inhibitor, or PBS as control. Following culturing for 16 hours, the caspase-3 activity in these kinds of cells was measured with the caspase assay system (Promega, Madison, WI, USA), using a spectrophotometer U-2000 (HITACHI, Tokyo, Japan) according to the manufacturer's instructions.

### 2.6. Test of ESA Binding to the Cells

An amount of 1.22 mg/mL ESA was fluorescently labeled by addition of 1 mg/mL Rhodamine 6G (Rh6G) in 0.15 M sodium carbonate buffer (pH = 9.0), followed by removal of free FITC by using a PD-10 column (GE Healthcare, CT, USA). OST cells and LM8 cells, suspended at a concentration of 2.0 × 10^5^ cells/mL, were cultured in 10% FBS ERDF medium. After 16 hours, the culture medium was exchanged with a culture medium containing 10% FITC-ESA solution, both types of cells were separately incubated for 3, 6, 9, 12, and 24 hours in a CO_2_ incubator at 37°C, respectively.

After the incubation, both cells were washed with cold PBS twice. Both cell suspensions were then analyzed by flow cytometry using a FACS Calibur instrument (Becton Dickinson, Mansfield, MA, USA). In a similar way, the binding activities of ESA (labeled with either rhodamine 6G (Rh6G) or FITC) to the sugar chains on the surface of OST cells were examined by incubation with *α*-mannnosidase, or *β*-mannnosidase, or endoglycosidase H for 2 hours before adding fluorescenctly labeled ESA. After incubation for 1 hour, the ESA binding to the OST cells was evaluated by using a fluorescence microscope (BH2-RFC, Olympus Corp, Tokyo, Japan) and the flow cytometer.

### 2.7. Preparation of a Lipidic ESA-Conjugate

ESA-SuPE, a phospholipid-ESA conjugate, was prepared as follows: 100 *μ*L of a SuPE solution (1.25 mg/mL in chloroform) were added to a test tube. A thin film of SuPE formed after evaporation of chloroform under a stream of nitrogen gas. Afterwards, 2.5 mL of an ESA solution (0.675 mg/mL) were added to the film to react with SuPE in 0.15 M sodium carbonate buffer (pH 9.0) at room temperature. The reaction mixture was incubated for 2 hours with vortexing for a few seconds every 30 min, followed by letting the suspension stand at 4°C overnight. Residual SuPE in the buffer solution was removed by gel filtration with a PD-10 column packed with Sephadex G-25 (GE Healthcare; Buckinghamshire, England).

### 2.8. Preparation of Different Types of Span 80 Vesicles

In the present work, four types of Span 80 vesicles were prepared. Type 1: Span 80 vesicles with immobilized ESA and immobilized PEG (EPV), containing as inner aqueous solution PBS. Type 2: Span 80 vesicles (called “control vesicles”: CV) containing encapsulated FITC. Type 3: Span 80 vesicles with immobilized ESA (EV) containing encapsulated FITC. Type 4: Span 80 vesicles with immobilized ESA and immobilized PEG (EPV) containing encapsulated FITC. The vesicles of types 2, 3, and 4 contained a 0.15 M sodium carbonate buffer solution (pH = 9.0) containing 1 mg/mL FITC as inner aqueous solution.

The vesicles were prepared with the two-step emulsification method in pretty much the same way of as described in the previous papers [6, 19]. In this work, some minor modifications were applied for the preparation of EPV containing FITC. A volume of 0.6 mL of the inner aqueous solutions (the sodium carbonate buffer solution containing FITC as mentioned above) was added to 6 mL of a n-hexane solution containing Span 80 (264 mg), purified lecithin (24 mg) and cholesterol (12 mg), followed by the first emulsification for 6 min at 17,500 rpm using a micro-homogenizer NS-310E 2 (Microtec Co., Ltd., Funabashi, Japan). Afterwards, the solvent was removed in a rotary evaporator at 28°C under reduced pressure, yielding a water-lipid emulsion to which 6 mL of the ESA-SUPE solution (obtained as described above) containing Tween 80 (96 mg) and DSPE-PEG_2000_ (14.2 mg/mL) were added, followed by the second emulsification with the homogenizer for 2 min at 3500 rpm to obtain a heterogeneous Span 80 vesicle suspension. After stirring with a magnetic stirrer for 3 hours at room temperature, the vesicle suspension was stored overnight at 4°C. The vesicles were then purified by ultracentrifugation (50,000 rpm at 4°C for 120 min) in a Himac centrifuge CR15B (Hitachi Koki Co., Ltd., Tokyo, Japan). The lower phase was filtrated through 100-nm nucleopore track-etch polycarbonate membranes (Avanti Polar Lipids; Alabaster, AL, USA) and purified by gel filtration on a 7 cm (diameter) × 50 cm (length) column containing Biogel-A5m (Bio-Rad Laboratories, Richmond, CA, USA). CV containing FITC and EV containing FITC were also prepared in the same manner as above, but without both ESA and PEG (for CV), and without DSPE-PEG_2000_ (for EV), respectively. The diameters of CV, EV, and EPV, which contained FITC were 104 ± 7 nm, 100 ± 2 nm, and 103 ± 5 nm, respectively.

### 2.9. Analysis of the Binding of EPV to OST Cells

OST cells were inoculated in 6-well culture plates at a cell density of 2.0 × 10^5^ cells/mL suspended in D-MEM with 10% FBS. The cells were incubated for 16 hours. Afterwards, the culture medium was exchanged with 1.8 mL D-MEM containing 10% FBS and 0.2 mL PBS, CV containing encapsulated FITC, EV containing encapsulated FITC, or EPV containing encapsulated FITC. The cells were then kept for 15 min in a CO_2_ incubator at 37°C. After incubation, the OST cells were washed with cold PBS twice, followed by flow cytometric analysis.

## 3. Results

### 3.1. Effect of ESA on the Viabilities of OST Cells and LM8 Cells

**Figure 1:**
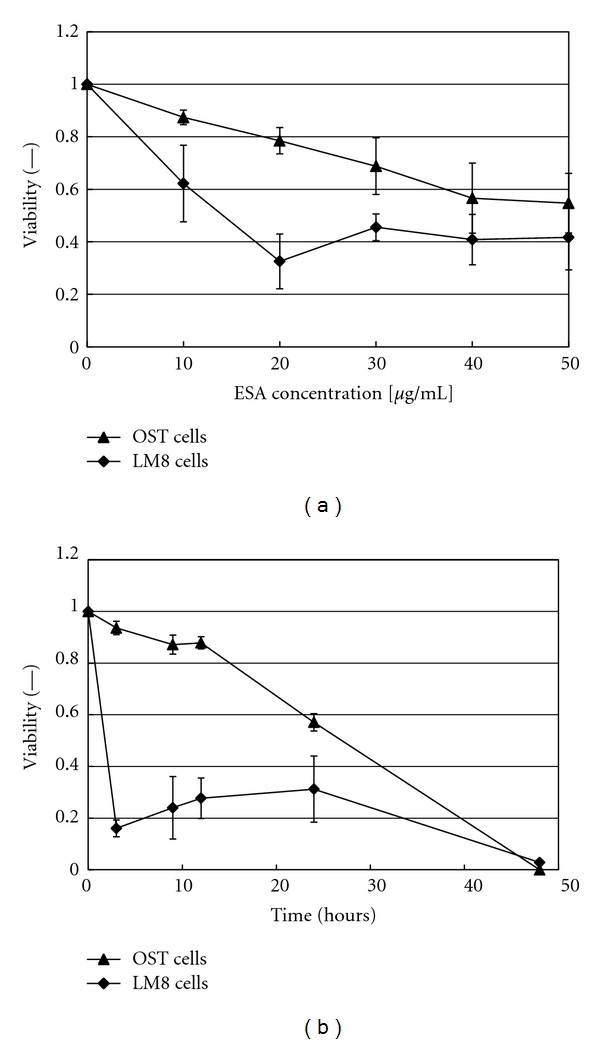
Cytotoxic effect of ESA on either OST cells or LM8 cells, as evaluated by means of propidium iodide staining. (a) Variation of the cell viability with increasing ESA concentration during incubation for 24 hours. (b) Time courses of the cell viabilities for [ESA] = 50 *μ*g/mL. For both set of data, mean values and standard deviations for three separate measurements are shown.

The viabilities of OST cells and LM8 cells were measured in the concentration range from 10 *μ*g/mL to 50 *μ*g/mL to evaluate the possible anticancer activity of ESA. As shown in Figure 1(a), the proliferations of both osteosarcoma cell types were inhibited by ESA. The inhibitory effect against the cell viability increased with increasing amounts of added ESA. Addition of 50 *μ*g/mL ESA, for example, decreased the cell viabilities of OST cells and LM8 cells to 54.7 ± 11.4% and 41.7 ± 12.3%, respectively. Furthermore, Figure 1(b) shows that the cell viabilities decreased with increasing elapsing time. The cell proliferation was inhibited completely by the addition of 50 *μ*g/mL ESA after incubation for 48 hours. These experiments clearly demonstrate the anticancer activity of ESA in the case of these osteosarcoma cells.

### 3.2. Apoptosis Induction by ESA in Both OST Cells and LM8 Cells as Determined by Means of a Double Staining Test

Previously, we have already demonstrated that ESA induces apoptosis in carcinoma cells [4]. The findings presented above about the inhibition of sarcoma cell proliferation (see Section 3.1.) suggested that ESA may also induce apoptosis in sarcoma cells. Therefore, apoptosis induction in either OST cells or LM8 cells by ESA was examined by means of the double staining test for Annexin V-PE and 7-ADD.

**Figure 2:**
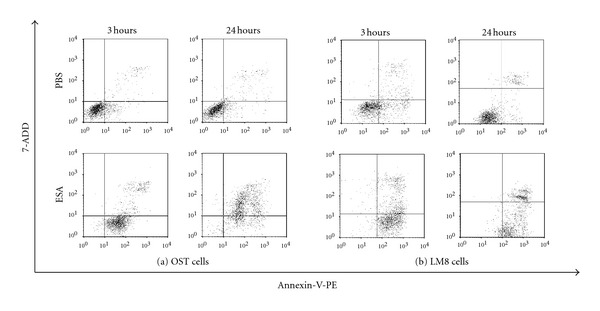
Apoptotic induction in either (a) OST cells or in (b) LM8 cells after adding ESA. The cells were cultured in 10% FBS D-MEM with 50 *μ*g/mL ESA (bottom panel). As control, only PBS (no ESA) was added (top panel). The cells were incubated with ESA for either 3 hours or 24 hours. Induction of apoptosis in these cells was detected by means of the double staining assay for annexin V-PE and 7-ADD.

The numerical values obtained from this analysis are displayed in Figure 2 and summarized in Table 1. As shown in Figure 2(a) and Table 1, the relative amount of cells in the lower right part of the diagram (indicating early stages of apoptosis) was 74.8% at an elapsing time of 3 hours after adding ESA, while in the case of the control cells (PBS-treated only, no ESA), the amount of the cells was 14.2% in the same part. Moreover, the amount of cells in the upper right part of the diagram (indicating dead cells) increased from 22.5% (at 3 hours after ESA addition) to 71.0% (at 24 hours). These results clearly show that ESA induced apoptosis in OST cells.

The same double staining test was also performed with LM8 cells. The results are also shown in Figure 2(b) and Table 1. The amount of cells in the lower right part of the diagram increased from 19.8% (control) to 68.2% at an elapsing time of 3 hours after adding ESA, being similar to the case of OST cells. The amount of cells in the upper right of the diagram also increased from 17.9% (at 3 hours) to 23.1% (at 24 hours). Thus, ESA also induced apoptosis in LM8 cells.

From the results in Sections 3.1 and 3.2, it was found that ESA specifically binds to OST cells and to LM8 cells, both being osteosarcoma cell lines, followed by induction of apoptosis. In the following investigations we mainly focused on OST cells, although some experiments were also carried out with LM8 cells.

### 3.3. Caspase-3 Assay in OST Cells after Adding ESA

**Figure 3:**
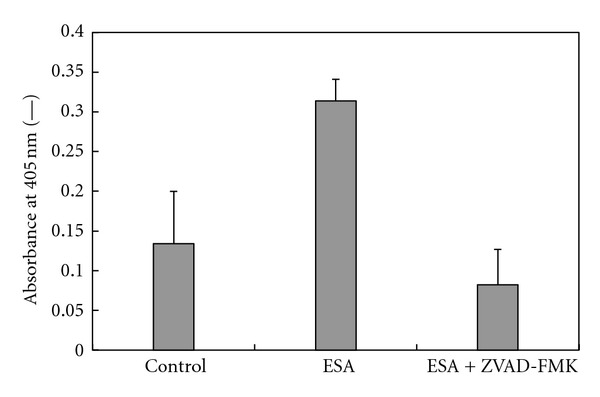
Determination of the caspase-3 activity of OST cells treated with ESA. The OST cells were cultured during 16 hours in D-MEM containing either a solution of 10% FBS and 50 *μ*g/mL ESA in PBS or a solution of 50 *μ*g/mL ESA and ZVAD-FMK (as caspase inhibitor) in PBS. Caspase-3 activity was determined from the absorbance values measured at 405 nm as “activity index” by use of a spectrophotometer. The values are means and standard deviations for three separate measurements.

The activity of caspase-3 in OST cells was measured by using the caspase-3 assay in combination with the caspase-3 inhibitor ZVAD-FMK, as outlined in Section 2.5. The values reported on the *y*-axis of Figure 3 are proportional to the amount (i.e., the activity) of expressed caspase-3, arising from the induced apoptosis in the OST cells. Upon addition of ESA, a 2.3-fold increase in caspase-3 activity was observed in comparison with the control (without ESA: only PBS). On the other hand, the addition of ZVAD-FMK inhibited the expressed capase-3 to almost the same level as in the case of the control. These data indicate that ESA induces apoptotic cell death in OST cells, which confirms the independent results presented in Figure 2.

### 3.4. Examination of the Binding of ESA to OST Cells and to LM8 Cells by Flow Cyotometric Measurements

**Figure 4:**
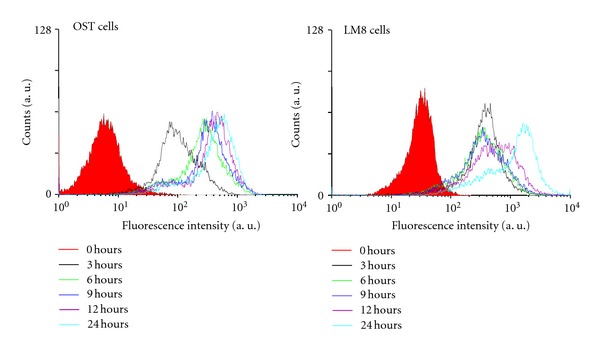
Specific binding of ESA to either OST cells or LM8 cells, as measured by using a flow cytometer. The cells were cultured with 10% FBS D-MEM containing FITC-labeled ESA at 37°C in a humidified atmosphere of 5% CO_2_. After incubation for 0, 3, 6, 9, 12, and 24 hours, the cells were washed with PBS, followed by evaluation of the amount of ESA which was bound to the cells.

To investigate the binding of ESA (labeled with FITC) to both OST cells and LM8 cells, flow cyotometric measurements were performed. As shown previously [4], ESA hardly binds to normal cells. If ESA-FITC binding to cells occurs, a rightward shift of the flow cyotometric curve is expected. This, indeed, was observed in the experiments with OST cells and LM8 cells, as shown in Figure 4. The fluorescence intensity of the cells treated with ESA-FITC increased significantly, as compared to the control cells (treated with PBS only). The curve shifts became larger with longer cell-incubation times: with both cell types, the shifts after 12 hours of incubation were larger than the shifts observed after 3 hours. This demonstrates binding of ESA-FITC to both cell types.

### 3.5. Fluorescence Microscopic Observation of the Binding of ESA to OST Cells That Were Pretreated with Glycosidases

In a previous study it was shown that ESA is a lectin that specifically binds to high-mannose type (HM) *N*-glycans [5]. The binding of ESA to OST cells that were pretreated with glycosidases was investigated by labeling cell-bound ESA with rhodamine 6G (Rh6G), see Section 2.6. 

**Figure 5:**
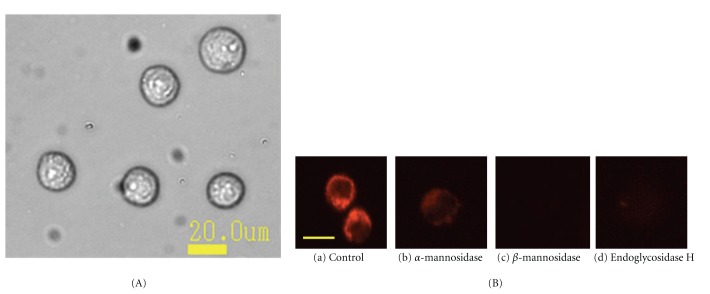
(A) Bright field image of OST cells. The diameter of the OST cells was 19.9 *μ*m ± 1.5 *μ*m. (B) Fluorescence microscopic observations of the binding of ESA to OST cells. The cells were pretreated for 2 hours with different glycosidases and then incubated with rhodamine 6G-labeled ESA. (a) Untreated cells (as control); (b) pretreated with *α*-mannosidase; (c) pretreated with *β*-mannosidase; and (d) pretreated with endoglycosidase H. After the pretreatment with the glycosidases, which led to a cleavage of some of the sugar chains on the surface of the OST cells, incubation of the pretreated cells with rhodamine 6G-labeled ESA occurred during 1 hour at 37°C in a humidified atmosphere of 5% CO_2_. Scale bar shows approximately 20 *μ*m. This scale was calculated from the bright field image.

First, the OST cells were pretreated with glycosidases to cleave sugar chains on the cell surface. Incubation was for 2 hours using one of the following three glycosidases, *α*-mannnosidase, *β*-mannnosidase, or endoglycosidase H. The method of Rh6G labeling with ESA was performed by incubating ESA with Rh6G as mentioned in Section 2.6. Then, the ESA labeled with Rh6G was bound to the cells by incubating the cells for 1 hour, followed by a fluorescence microscopic observation of the labeled cells. As shown in Figure 5, non-treated OST cells (as control) displayed Rh6G fluorescence, but other OST cells that were pretreated with a glycosidases showed almost no fluorescence. This means that ESA could not recognize the molecular structure of the sugar-chains on the surface of OST cell that were cleaved by glycosidases; ESA only recognized the native structure of the sugar-chains of the OST cells. Thus, with these experiments it could be demonstrated that ESA specifically binds to OST cells, through recognition of the sugar chains on the surface of the cells.

### 3.6. Flow Cytometric Analysis of the Specific Binding of ESA to OST Cells Treated with Glycosidases

**Figure 6:**
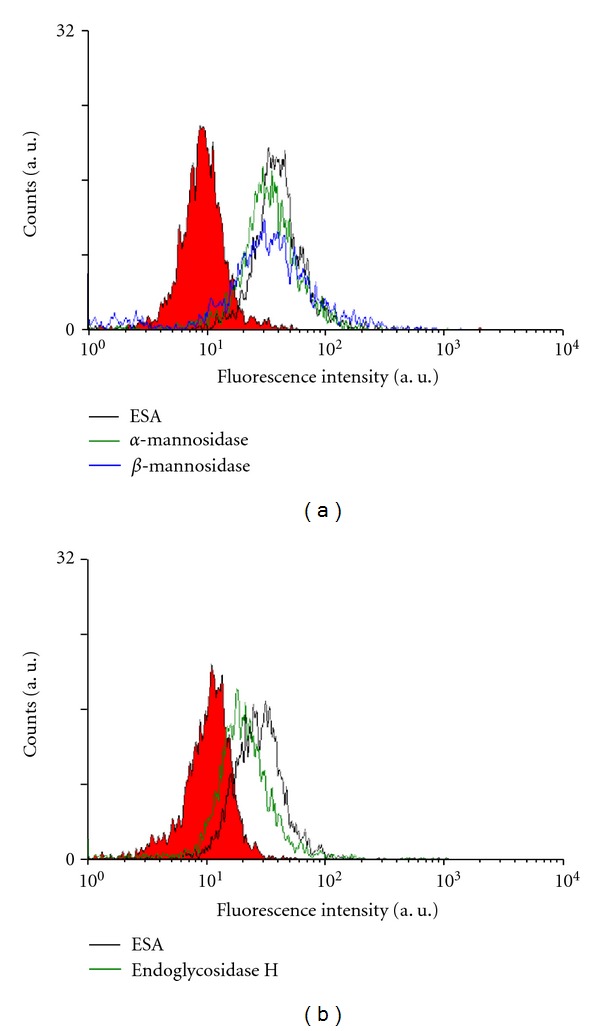
Flow cytometric analysis of OST cells that were pretreated with a glycosidase as described in the legend of Figure 5 and then incubated with FITC-labeled ESA (balck line). Pretreatment was with either *α*-mannosidase (green line) or with *β*-mannosidase (blue line) (a), or with endoglycosidase H (green line) (b). The filled curves represent control measurements with untreated cells. PBS was added to OST cells as control (red fill).

To confirm the specific binding of ESA to OST cells, a flow cytometric examination was also performed in a similar way as described in Sections 3.4 and 3.5. The results are shown in Figure 6(a) for cells treated with *α*-mannosidase and *β*-mannosidase, and in Figure 6(b) for cells treated with endoglycosidase H. In both cases, the decreases in fluorescence intensity in those cells that were treated with a glycosidase, if compared to untreated cells, were obvious. The intensity decrease in the case of treatment with *α*-mannosidase seemed to be smaller than in the case of *β*-mannosidase or endoglycosidase H. This is in good agreement with the images shown in Figure 5 obtained with an independent analysis. Weak Rh6G fluorescence was detectable in glycosidase-treated OST cells—although with rather low intensity—only if the treatment was with *α*-mannosidase. In the other two cases, there was no detectable fluorescence (Figure 5).

### 3.7. Possible Application of ESA as Sarcoma-Targeting Ligand Immobilized on Span 80 Vesicles

**Figure 7:**
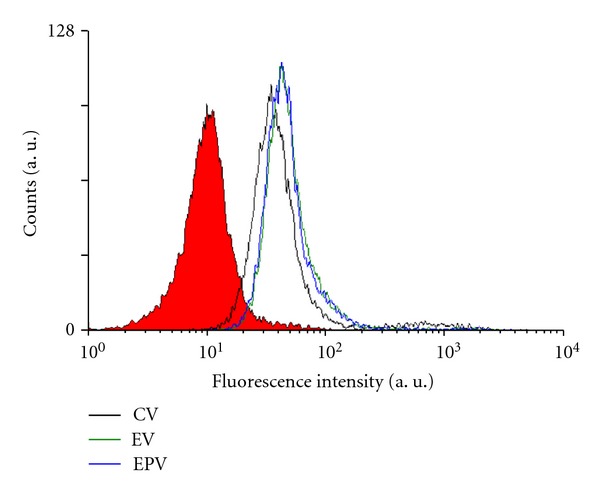
Flow cytometric analysis of the interaction between OST cells and different types of Span 80 vesicles containing entrapped FITC: control vesicles (CV, black line), vesicles with immobilized ESA (EV, green line), and PEGylated vesicles with immobilized ESA (EPV, blue line). Before analysis, the OST cells were incubated with the vesicles during 15 min at 37°C in a humidified atmosphere of 5% CO_2_. PBS was added to OST cells as control (red fill).

To test whether ESA could be used as osteosarcoma-targeting ligand on a vesicular DDS, Span 80 vesicles with surface bound ESA were prepared, and the interaction of these vesicles with OST cells was compared with the interaction of vesicles that did not have surface bound ESA. Three types of Span 80 vesicles were prepared and tested (see Section 2.9): CV (control vesicles, no ESA), EV (vesicles with immobilized ESA), and EPV (PEGylated vesicles with immobilized ESA). All these vesicles contained encapsulated FITC as fluorescent probe. The vesicles were then mixed with OST cells and incubated, as mentioned in Section 2.9. Then, flow cytometric measurements were performed. As shown in Figure 7, the fluorescence intensity in both cases was higher than for cells treated with CV containing FITC. This means that both types of vesicles with surface bound ESA, EPV, and EV bind to OST cells stronger than CV does. Furthermore, the fluorescence intensity of the cells treated with EPV containing FITC was found to be almost equal to the fluorescence intensity of the cells that were treated with EV containing FITC. Therefore, PEGylation did not hinder the binding of ESA to the sugar chains on the surface of the cells. Thus, Span 80 vesicles with immobilized ESA may be well suited for the development of a DDS for targeting osteosarcoma cells.

### 3.8. Cytotoxic Effects of EPV against OST Cells

**Figure 8:**
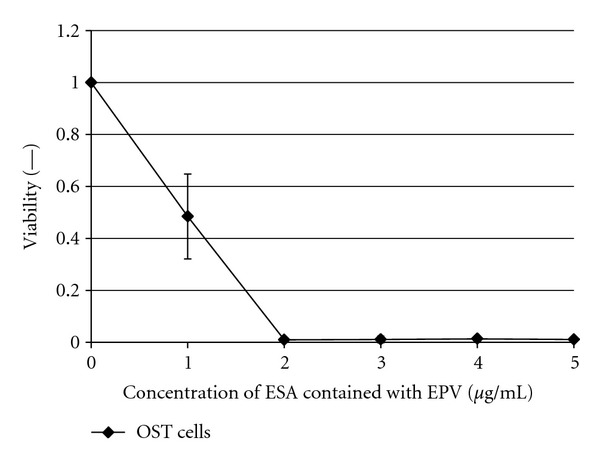
Cytotoxic effect of ESA in EPV on OST cells. The cell viability was evaluated with the propidium iodide staining. The OST cells were incubated during 48 hours at 37°C with EPV at the given concentration in D-MEM containing 10% FBS in a humidified atmosphere of 5% CO_2_. Mean values and standard deviations for three separate measurements are plotted.

In a final investigation, the anticancer activity of EPV against osteosarcoma cells was examined *in vitro*. The variation of the OST cells viability as a function of the concentration of added ESA (incubation time was 48 hours) is shown in Figure 8. EPV also clearly showed a strong anticancer activity against OST cells, inhibiting proliferation of OST cells completely in a culture medium that contained 2 *μ*g/mL ESA. This result is promising as it shows that PEGylated Span 80 vesicles with immobilized ESA are potentially useful as drug carrier system with endogenous antitumor activity against osteosarcoma. In the ESA concentration range above about 2 *μ*g/mL complete death of the OST cells was observed, as shown in Figure 8. This demonstrates that EPV not only can function as targeting unit (see Section 3.7), but also efficiently inhibit OST cell growth.

## 4. Discussion

It is known that the carbohydrate structures vary among the different cancer cell lines [27, 28]. In this work, we report about our findings that ESA has anticancer activity not only against carcinoma [4] but also against sarcoma. This conclusion is based on the observation that both types of osteosarcoma cells, OST cells and LM8 cells, were significantly destroyed if incubated with ESA at a concentration of 50 *μ*g/mL during a period of 24  hours, as shown in Figure 1(a), and also destroyed completely if during 48 hours, as shown in Figure 1(b).

The effect of ESA on the viabilities of osteosarcroma cells was compared with the effect of ESA carcinoma cells studied previously [4], see S-2, Supplementary Material, available online at doi:10.1155/2012/842785. The Supplementary Material contains (i) data on the cytotoxicity and binding affinity of free ESA and EV for normal cells and for cancer cells; and (ii) a comparison of the effect of free ESA on the cell viabilities of osteosarcoma and carcinoma cells. This comparison indicates that the antiproliferative activity of free ESA in sarcoma cells is higher than in carcinoma cells, which may be related to differences in the carbohydrate structure of the surface of the two cell types. This point needs to be investigated further.

We already reported [4] that ESA specifically binds to high mannose type sugar chains in the case of carcinoma cells, inducing apoptotic cell death. As shown in Figure 4 of the flow cytometric measurements, it was confirmed that ESA bound not only to carcinoma cells but also to sarcoma cells like OST cells and LM8 cells. Moreover, pretreatment of OST cells with different types of glycosidases, which cleaved the sugar chains on the surface of the OST cells, significantly decreased the binding of ESA to the cells (Figures 5 and 6). These results provide evidence that binding of ESA to the sarcoma cells occurs through specific interactions between ESA and carbohydrate chains on the cell surface. ESA exhibited higher affinity towards OST cells as compared to LM8 cells (Figure 4). The reason for this may be due to differences in the carbohydrate structure in the two cell types. This point needs to be also investigated, however, before any clear conclusion about the cell specificity can be drawn.

ESA induces apoptosis in osteosarcoma cells as shown by using the double staining test for Annexin-V and 7-ADD [25–27]. At an elapsing time of 3 hours after adding ESA, apoptosis in both OST cells and LM8 cells was obvious. Moreover, almost all of the OST cells were dead after 24 hours incubation with ESA (50 *μ*g/mL), as shown in Figure 2(a). The number of LM8 cells appearing in the upper right region of the plot did not seem to increase (see Figure 2(b)). This apparent failure in staining is related to the apoptotic progress of the cell, and the apoptosis couldn't be correctly measured with the double staining method. In fact, in the analysis of the flow cytometry, in the LM8 cells often fragmented and therefore counted correctly, when incubated during 24 hours with ESA (data not shown).

Induction of apoptosis in OST cells by ESA was demonstrated by measuring the expression of caspase-3 (see Figure 3). It was shown that the addition of ESA to OST cells led to apoptosis in cells of sarcoma, because the caspase-3 expression is known to be directly related to the apoptosis mechanism [29]. Thus, ESA may be used as efficient tumor-targeting ligand and apoptosis inducer in a DDS in a sarcoma therapy. As shown in our previous work, PEGylated Span 80 vesicles with immobilized ESA (abbreviated as EPV) are rather promising drug carriers for the treatment of carcinoma cancers [6]. Therefore, the use of EPV may be expanded to the treatment of sarcoma.

The ability of ESA, and EPV, as targeting unit and apoptosis inducer in the case of cells of sarcoma was examined further by flow cytometry as well as cell viability measurements, choosing OST cells as typical sarcoma cell type. As shown with the flow cytometric measurements in Figure 6, targeting of ESA to OST cells *in vitro* was observed from the shift of the flow cytometric curve to the right hand side (see Figure 6). Furthermore, comparing EPV with CV in Figure 7 (as mentioned in Section 3.7.), it was found that the macromolecular structure of PEG on the vesicle surface did not hinder OST cell binding of ESA which was localized on the vesicle surface together with PEG. This is a very important phenomenon. It may be due to the high mobility of both ESA and PEG, because of the high membrane fluidity of Span 80 vesicles, as mentioned previously [19, 30]. Therefore, the use of Span 80 vesicles as DDS is very effective. In addition, EPV showed anticancer activity against OST cells since after an elapsing time of about 48  hours after the addition of EPV at an ESA concentration of 2 *μ*g/mL, the OST cell viability was reduced to almost zero, as shown in Figure 8. It seems that the anticancer activities of ESA against OST cells in the vesicle system (Figure 8) is stronger than those in free ESA system (Figure 1). However, the activities of the two systems cannot be compared directly, because either the incubation time or the ESA concentration was different in the two systems. For example, for a direct comparison of the activities of the two systems against OST cells, the time-course of the viability upon addition of free ESA system (Figure 1(b)) should be measured at [ESA] = 2 *μ*g/mL; at this concentration and after an incubation time of 48  hours, the cells were no more viable if the vesicles system was used (Figure 8). Unfortunately, the data obtained from measurements with free ESA at this low concentration showed great variations. 

On the other hand, we have already examined [4, 6] the cytotoxicity of either ESA or EV for various carcinoma cancer cells and normal cells, followed by examining the binding affinities of ESA and EV to the cells. In these experiments, Colo201 (human colon adenocarcinoma), MCF-7 (human breast adenocarcinoma), HeLa (human cervix adenocarcinoma), and HB4C5 cells (human hybridoma cell line) were used as carcinoma cells, and MCF10-2A (non-tumorigenic epitherial cell line) and normal fibroblasts (from the umbilical cord) were also used as normal cells. ESA and EV showed cytotoxicity against carcinoma cells but not against normal cells, see S-1, Supplementary Material.

**Figure 9:**
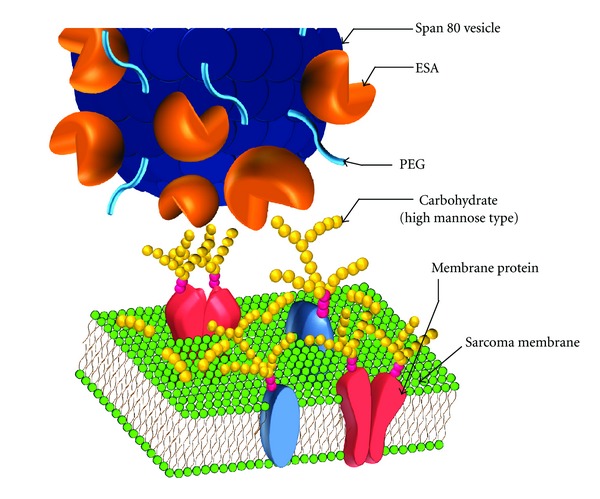
Graphical imaginary view indicating the binding between carbohydrate chains of high mannose type on sarcoma membrane and the ESA on the PEGylated Span 80 vesicle.

Figure 9 is a graphical imaginary view indicating the binding between carbohydrate chains of high mannose type on sarcoma membranes and ESA on the PEGylated Span 80 vesicle.

## 5. Conclusions

In the study presented, the following main results were obtained: (i) ESA specifically binds to sarcoma cells and induces apoptotic death of the cells; (ii) the antiproliferative activity of ESA in sarcoma is higher than the activity in carcinoma; (iii) ESA immobilized onto PEGylated Span 80 vesicles (EPV) shows antitumor activity against OST cells without any entrapped antitumor agents. Furthermore, in a previous study, it was already revealed that ESA and EV (ESA-immobilized on Span 80 vesicles) hardly bind to normal cells (either MCF10-2A (non-tumorigenic epithelial cells) or normal fibroblasts from the umbilical cord); and cytotoxicity caused by ESA and EV was not observed for these normal cells. Therefore, ESA has considerable potential as novel type of targeting ligand against sarcoma.

Based on all these findings, we propose using EPV as possible DDS not only for the targeted treatment of carcinoma, but also for the targeted treatment of sarcoma. Furthermore, the administration of PEGylated Span 80 vesicles with immobilized ESA, in which anticancer drugs are encapsulated, is expected to express more effective antitumor activity against sarcoma as compared to empty EPV.

We already performed first *in vivo* experiments by using either EV or EPV with entrapped anticancer drugs toward the development of a sarcoma therapy. The results will be presented in a separate paper.

## Supplementary Material

The Supplementary Material contains (i) data on the cytotoxicity and binding affinity of free ESA and EV for normal cells and for cancer cells; and (ii) a comparison of the effect of free ESA on the cell viabilities of osteosarcoma and carcinoma cells.

## Figures and Tables

**Table 1:** Cell state dependence on elapsing time after the addition of ESA to OST cells or LM8 cells. The cell states are classified as “living”, “apoptotic” or “dead”, as obtained from the data shown in Figure 2.

Elapsing time (h)	OST cells			LM8 cells		
	living (%)	apoptotic (%)	dead (%)	living (%)	apoptotic (%)	dead (%)
3	2.5	74.8	22.5	10.3	68.2	17.9
24	3.5	24.1	71.0	7.9	68.8	23.1

Please note that cells appearing in the upper left part of the diagram in Figure 2 (Annexin V-PE negative and 7-ADD positive) are not included in the table.
