# Supplementary material for: Active Targeting to Osteosarcoma Cells and Apoptotic Cell Death Induction by the Novel Lectin Eucheuma serra Agglutinin Isolated from a Marine Red Alga
Source: J Drug Deliv. 2012 Dec 27;2012:842785. doi: 10.1155/2012/842785 (PMC3543805; doi:10.1155/2012/842785)
Supplement: Supplementary file 1 — The Supplementary Material contains (i) data on the cytotoxicity and binding affinity of free ESA and EV for normal cells and for cancer cells; and (ii) a comparison of the effect of free ESA on the cell viabilities of osteosarcoma and carcinoma cells. [file 842785.f1.pdf]

# Supplementary Material

# Active targeting to osteosarcoma cells and apoptotic cell death induction by the novel lectin *Eucheuma serra* agglutinin (ESA) isolated from a marine red alga

Keita Hayashi[a], Peter Walde[b,2], Tatsuhiko Miyazaki[c], Kenshi Sakayama[d], Atsushi Nakamura[d], Kenji Kameda[e], Seizo Masuda[e], Hiroshi Umakoshi[a] , Keiichi Kato[f,1]

[a]Division of Chemical Engineering, Graduate School of Engineering Science, Osaka University, 1-3 Machikaneyama-cho, Toyonaka, Osaka 560-8531, Japan

[b]Department of Materials, ETH, Wolfgang-Pauli-Strasse 10, CH-8093 Zürich, Switzerland

[c]Department of Pathogenomics, Graduate School of Medicine, Ehime University, Shitsukawa, Toon, Ehime 791-0295, Japan

[d]Department of Bone and Joint Surgery, Graduate School of Medicine, Ehime University, Shitsukawa, Toon, Ehime 791-0295, Japan

[e]Integrated Center for Science Shigenobu Station, Ehime University, Shitsukawa, Toon, Ehime 791-0295, Japan

[f]Department of Materials Science and Biotechnology, Graduate School of Science and Engineering, Ehime University, 3 Bunkyo-cho, Matsuyama, Ehime 790-8577, Japan

[1] First corresponding author. TEL.: +81 976 4686; fax: +81 976 4686

[2] Second corresponding author. TEL.: +41 44 6320473; fax: +41 44 6321265

*E-mail address:* keiichi1017@mc.pikara.ne.jp (K Kato), peter.walde@mat.ethz.ch (P Walde)

**S-1 Cytotoxicity and binding affinity of either free ESA or Span 80 vesicles immobilized ESA (EV) for normal cells and cancer cells**

We have already reported [4,6] about the cytotoxicity of either ESA or **EV (**Span 80 vesicles immobilized ESA) for various carcinoma cells and for normal cells, followed by examining the binding affinities of ESA and **EV** to the cells. In these previous studies Colo201 (human colon adenocarcinoma), MCF-7 (human breast adenocarcinoma), Hela (human cervix adenocarcinoma) and HB4C5 (human hybridoma cell line) were used as carcinoma cells, and MCF10-2A（non-tumorigenic epithelial cell line） and normal fibroblast（from the umbilical cord） were used as normal cells.

As a result, it could be demonstrated that (i) cytotoxicities of ESA and **EV** could hardly be observed for normal cells (MCF10-2A); (ii) while ESA and **EV** specifically bound to carcinoma of Colo201, MCF-7, Hela and HB4C5 cells, ESA and **EV** hardly bound to normal cells (either MCF10-2A or normal fibroblasts from the umbilical cord); (iii) the lower incidence of the binding of either ESA or **EV** to normal cells seems to correlate with a lower toxicity in the case of normal cells. The details are given below.

**S-1.1 Cytotoxicity of either free ESA or Span 80 vesicles immobilized ESA (EV) against normal cells and cancer cells**

**S-1.1.1 The cytotoxicity of free ESA**

**Figure S1** shows the comparison of the effect of free ESA on the cell viabilities of MCF10-2A (normal cells) and of Colo201 (carcinoma cells). The data indicate that cytotoxicity of ESA against MCF10-2A could hardly be observed, while ESA was highly cytotoxic in the case of Colo201 cells.

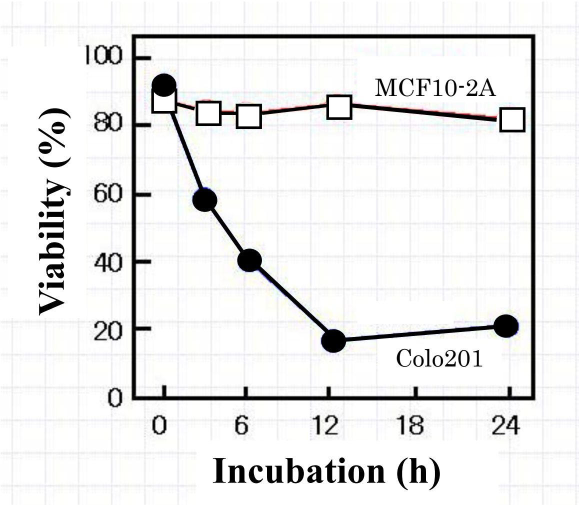


**Figure S1** Comparison of the effect of free ESA on the cell viability of MCF10-2A cells (normal cells) and on the viability of Colo201 cells (carcinoma cell); the cells were inoculated at $1.0 \times 10^5$ cells/ml in ERDF medium with 10% FBS and 12.8 μg/ml ESA. The cell number and the cell viability were measured with the trypan blue dye exclusion test on a hemocytometer. The values are means of two separate measurements.

### S-1.1.2 Cytotoxicity of EV

The cytotoxicity of **EV** (Span 80 vesicle immobilized ESA) was examined for MCF10-2A and Colo201, see [6]. It was found that the cytotoxicity of **EV** against MCF10-2A could be hardly observed, while strong cytotoxicity for Colo201 was found, similarly to the behavior of free ESA (see above).

## S-1.2. Binding ability of either free ESA or EV for normal cells and for cancer cell

### S-1.2.1 Cell binding ability of free ESA

**Figure S2** shows a comparison of the laser scanning confocal fluorescent microphotographs of Colo201 cells (as cancer cells) and MCF10-2A cells (as normal cells), which were both pretreated with ESA labeled with FITC. The FITC fluorescence could hardly be observed in the microphotographs of MCF10-2A, while the fluorescence could clearly be observed in the case of Colo201. From these results, ESA was revealed to be able to bind to Colo201 as cancer cell specifically, while binding to MCF10-2A did not occur.

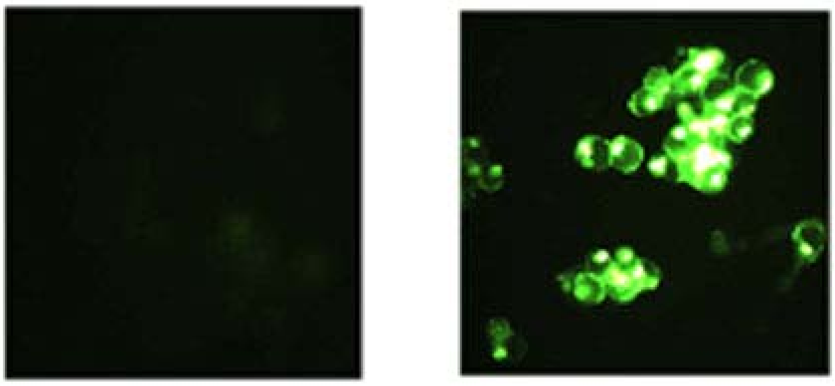

(a) MCF10-2A (b) Colo201

**Figure S2** Comparison of the laser scanning confocal fluorescent microphotographs of (a) MCF10-2A cells (as normal cell: non-tumorigenic epithelial cell line), and (b) Colo201 cells (as cancer cell: human colon adenocarcinoma), which were both incubated in ERDF medium supplemented with ESA labeled with fluorescent FITC for 3 hrs. The cells were observed after the incubation by a laser scanning confocal fluorescent microscope.

### S-1.2.2 Cell binding ability of EV

Binding experiments were carried out by using two kinds of normal cells, MCF10-2A and normal fibroblasts, and using Colo201, MCF-7 and Hela and HB4C5cells as cancer cells. Cancer-specific binding of Span 80 vesicles immobilized ESA (**EV**) was examined. **EV** encapsulating FITC were added to the culture medium of each cell line (Colo201, MCF-7. Hela, HB4C5, MCF10-2A and normal fibroblasts), and cultured for 3 h. Afterwards, the cells were washed with cold PBS, and the FITC intensity in the cells was analyzed by flow cytometry, see [4] for details. **EV** was found to specifically bind to the cancer cells (Colo201, MCF-7. Hela, and HB4C5), but not to the normal cells (MCF10-2A and normal fibroblasts), smililarly to what was observed in the experiments with free ESA labeled with FITC.

**S-2 Comparison of the effect of free ESA on the cell viabilities of osteosarcroma and carcinoma cells**

The effect of ESA on the viabilities of osteosarcroma cells was compared with the effect of ESA on the viabilities of carcinoma cells, as previously determined [4]. MCF-7 cells and Hela cells were used as carcinoma cells [4], and OST cells and LM8 cells (this work) were used as a sarcoma cells. At the same concentration conditions of free ESA (50 μg/ml), the viabilities of the carcinoma cells (elapsed time = 72 hours, [4]) were estimated, followed by comparing with the viabilities of the sarcoma cells (elapsed time = 24 hours or 48 hours, this work). The data are shown in **Table S1** and indicate that the antiproliferative activity of free ESA in sarcoma cells is higher than the activity of free ESA in carcinoma cells, because the viabilities of the sarcoma cells (elapsed time = 72 hours) decreased stronger as compared to the viability decrease for the carcinoma cells, even in the case of the sarcoma cells the elapsed time was shorter (24 hours or 48 hours). The reason of the difference of the antiproliferative activity between carcinoma cells and sarcoma cells may be due to differences in the carbohydrate structure in the two cell types.

**Table S1** The effect of ESA on the viabilities of osteosarcroma cells (this work) in comparison with those on carcinoma cells (previous study, [4]

| Cells | Carcinoma [4] | | Sarcoma | | | |
|---|---|---|---|---|---|---|
| | MCF-7 | Hela | OST | | LM8 | |
| ESA conc. [μg/ml] | 50 | 50 | 50 | 50 | 50 | 50 |
| Elapsed time [h] | 72 | 72 | 24 | 48 | 24 | 48 |
| Viability [%] | 50 | 10 | 55 | 0 | 42 | 0 |

Note: The experimental error of the viabilities is roughly estimated to be within ±15%. The values for the viability of the carcinoma cells were calculated from the data in Fig. 1 of [4,] while the values for the sarcoma cells are calculated from the data given in Fig. 1 (this work).

**References**

[4] T. Sugahara, Y. Ohama, A. Fukuda, M. Hayashi, A. Kawakubo and K. Kato, "The cytotoxic effect of Eucheuma serra agglutinin (ESA) on cancer cells and its application to molecular probe for drug delivery system using lipid vesicles," *Cytotechnology*, vol. 36, no. 1-3, pp. 93-99, 2001

[6] Y. Omokawa, T. Miyazaki, P. Walde, K. Akiyama, T. Sugahara, S. Masuda, A. Inada, Y. Ohnishi, T. Saeki and K. Kato, "In vitro and in vivo anti-tumor effects of novel Span 80 vesicles containing immobilized Eucheuma serra agglutinin," *Int J Pharm*, vol. 389, no. 1-2, pp. 157-167, 2010
